# A Core Circuit Module for Cost/Benefit Decision

**DOI:** 10.3389/fnins.2012.00123

**Published:** 2012-08-31

**Authors:** Keiko Hirayama, Marianne Catanho, Jeffrey W. Brown, Rhanor Gillette

**Affiliations:** ^1^The Neuroscience Program, University of IllinoisUrbana, IL, USA; ^2^Electrical and Computer Engineering, University of IllinoisUrbana, IL, USA; ^3^Biophysics and Computational Biology, University of IllinoisUrbana, IL, USA; ^4^Department of Molecular and Integrative Physiology, University of IllinoisUrbana, IL, USA

**Keywords:** approach/avoidance, central pattern generator, neuroeconomics, neuronal switch, *Pleurobranchaea*, simulation, decision making

## Abstract

A simple circuit for cost-benefit decision derived from behavioral and neural studies of the predatory sea-slug *Pleurobranchaea* may closely resemble that upon which the more complex valuation and decision processes of the social vertebrates are built. The neuronal natures of the pathways in the connectionist model comprise classic central pattern generators, bipolar switch mechanisms, and neuromodulatory state regulation. Marked potential exists for exploring more complex neuroeconomic behavior by appending appropriate circuitry *in simulo*.

## Introduction

Organisms are designed to engage three basic functions: resource acquisition, defense against accident (e.g., predation and disease), and reproduction. Their lifestyles represent behavioral economic strategies, and range in complexity from very simple solitary foraging to the complicated, multi-layered economies of the social vertebrates. The complexities of valuation, decision-making, and lifestyles are parallel over this range. What are the gradations of complexity in behavioral economy, and how are they traversed in evolution? Let’s begin by examining the simpler systems.

In simple terms, goal-directed decision is regulated by an animal’s appetitive state, which is the summation of sensation, internal state, and learning. Operationally, the appetitive state itself is the likelihood that an animal will perform any of a repertory of goal-directed, homeostatic behaviors. The basic premise of behavioral economics is that decisions so made will, on average, optimize success in foraging and reproduction, and minimize accompanying risk. How these computations are effected at levels of neural networks and nerve cells is basic to understanding the genesis of behavioral economics in nervous system function.

For foraging animals, a most critical and simple behavioral decision regulated by appetitive state is that for approach or avoidance of a stimulus. The neuronal nature of appetitive state, and how it toggles decision, are problems that have been investigated in the predatory sea-slug, *Pleurobranchaea* *californica*.

## The Economic Landscape of a Simpler Predator

**Figure 1:**
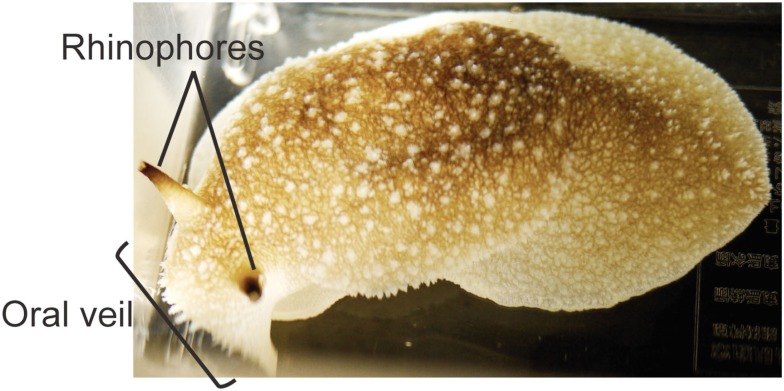
**A *Pleurobranchaea* with chemotactile oral veil and rhinophores indicated**.

To put the decision mechanism in natural context, it is useful to describe *Pleurobranchaea*’s simple economy of lifestyle. The sea-slug (Figure 1) is an opportunistic predator with simple behavior and nervous system, and it makes value-based decisions that balance need for resource against personal risk (Gillette et al., 2000). Very hungry animals not only have very low thresholds for feeding stimuli, but will even attack mildly noxious stimuli such as acidic seawater. Satiated animals actually actively avoid food stimuli, and partly satiated animals may avoid weak appetitive stimuli but attack stronger stimuli. Thus, level of effort is related to need (hunger), and the perceived value of a resource is weighed against the potential risk of an attack, such as in prey defenses and possible attraction of another predator (like a cannibal conspecific), and probable cost of energy outlay in an attack. The ability to associate specific odors with the positive or negative consequences of an attack on potential prey (Davis et al., 1980; Mpitsos and Cohan, 1986a,b) lends the predator another important skill for optimizing foraging success.

Thus, a simple cannibal predator like *Pleurobranchaea* operates at an extremely simple neuroeconomic level, one in which the three basic organismal functions are satisfied in an uncluttered manner. The model for decision, discussed below, is so simple that it may represent a basic core type of circuit whose relations are common to most foragers, and one onto which the more complex circuits for value and risk in social vertebrates are built in evolution.

## Background to the Present

**Figure 2:**
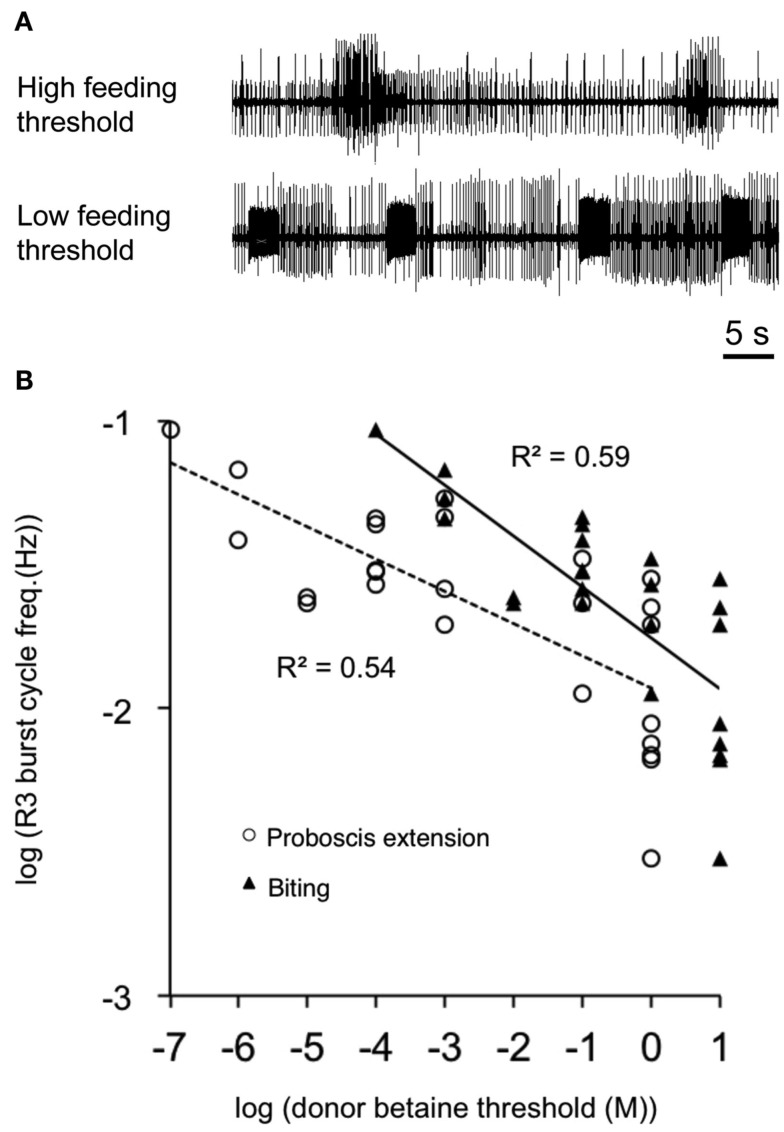
**Conservation of appetitive state in the isolated CNS**. Spontaneous feeding nerve burst frequency correlated with donors’ sensory feeding thresholds to the appetitive stimulant betaine (trimethylglycine). **(A)** Spontaneous burst frequency recorded from buccal motor nerve *R3* of isolated CNSs was less from high-threshold donors than from low-threshold donors. **(B)** *R3* burst frequency was an approximately linear function of donor feeding thresholds on a log–log plot (*n* = 25; *R*^2^ = 0.54 and 0.59 for proboscis extension and biting, respectively). Line fits were by least squares. Three high-threshold donor CNSs did not show burst patterns in *R3* and were excluded from the figure. From Hirayama and Gillette (2012).

Dr. Rimmon Fay, a notable biological supplier of southern California, was a key figure in the history of neuroethological research for several molluscan preparations, including *Aplysia*, *Navanax*, *Bulla*, and *Pleurobranchaea*. Without his supply side efforts in the 1960s to the 1980s, it is unlikely that much of the present progress in molluscan neuroethology would have been made. The strenuousness of his efforts was made clear to those of us lucky enough to accompany him on a collecting cruise. He noted a population boom of *Pleurobranchaea* and sent several specimens to researchers at Stanford University. Davis and Mpitsos (1971) realized the marked potential for a model system for study of behavioral choice, and the following decades saw novel reports on odor learning abilities for food avoidance (Mpitsos and Collins, 1978; Davis et al., 1980; Mpitsos and Cohan, 1986a,b) and demonstrations of diverse and actual neuronal mechanisms of choice involving network interactions for feeding vs. withdrawal to touch (Kovac and Davis, 1980a,b), escape vs. feeding (Jing and Gillette, 1995), escape vs. turning (Jing and Gillette, 2003). Behavioral studies showed that animals could integrate hunger state, taste, and pain to decide between approach and avoidance of appetitive stimuli, consistent with a cost-benefit decision mechanism rooted in appetitive state (Gillette et al., 2000). Study of this phenomenon was given a great boost by findings that the isolated CNS conserved the appetitive state of the intact donor (Hirayama and Gillette, 2012). Thus, spontaneous activity in the feeding network in CNS isolated from hungry animals was higher than for those less hungry. In fact, the spontaneous activity recorded in feeding motor nerves of isolated CNSs was proportionate to the feeding thresholds of CNS donors (Figure 2), in a remarkably linear log–log relationship.

A second helpful finding was that isolated CNSs display fictive turns, recorded in motor nerves following unilateral stimulation of sensory nerves that innervate the chemotactile oral veil (Jing and Gillette, 2003). The third finding was that the appetitive state of isolated CNS also controlled the direction of the fictive turn: turn direction was contralateral to the stimulated nerve in CNS from less hungry donors, but ipsilateral in those from hungry animals (Hirayama and Gillette, 2012). It was found that increasing the excitation state of the feeding network – either by driving an identified feeding command neuron or by stimulating a sensory nerve innervating the buccal cavity – could reversibly change fictive decision from an avoidance to an orienting turn. This observation had two implications: first, the turn network was probably organized by default for avoidance, and second, that corollary outputs from the feeding network must somehow switch sensory input from one side of the turn network to the other. This resembles control of vertebrate spinal reflexes, whose default circuits are redirected to other, even oppositely directed, behaviors by descending voluntary control (Sherrington, 1906; Stuart, 2002).

## The Core Module for Cost-Benefit Decision

**Figure 3:**
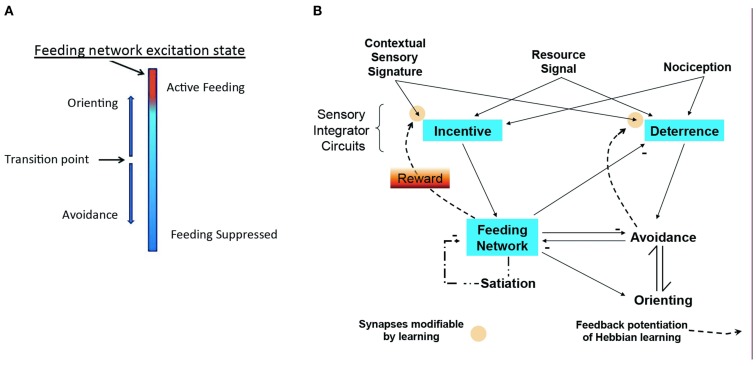
**Modeling homeostatic decision**. **(A)** Excitation state of the homeostatic feeding network switches avoidance to approach. **(B)** Sensory inputs for resource quality, sensory signatures, and nociception access sensory networks for *Incentive* and *Deterrence*, which promote excitation of feeding and avoidance turns, respectively. Excitation in the homeostatic network suppresses avoidance and promotes orienting turns (approach). Active avoidance and satiation inhibit the homeostatic network, while homeostatic network activity suppresses *Deterrence*. Modulatory feedback pathways from the *Feeding* and *Avoidance* networks potentiate learning of sensory signatures, mediating reward. Modified from Hirayama and Gillette (2012).

The model of Figure 3 emerged from the studies of the isolated CNS. It takes into account that the feeding motor network is basically a homeostatic neural network that economically combines representation of appetitive state with central motor pattern generation. To summarize the model: the excitation state of the feeding network embodies appetitive state as a sum of intrinsic excitability, stimulus salience, and effects of memory. Corollary outputs from the network toggle the directional response of the turn network to change avoidance to orienting. Adding known relations of sensory pathways and interactions among neuronal networks in *Pleurobranchaea* fills out a complete, simple model for decision. The model represents a basic cost-benefit decision module for foraging, encoding appetitive state in a homeostatic neuronal network that controls decision for approach/avoidance to appetitive and noxious stimuli.

The model is built on an empirical approach to a general theory of cost-benefit decision. It is accessible to hypothesis testing and modifiable from the results of those tests. It represents three types of neuronal networks interconnected in feed-forward and feedback loops: (1) The goal-directed feeding network is regulated internally by satiation state, and externally by sensory inputs that include effects of odor memory (Davis and Gillette, 1978). The feeding network makes coordinating connections with agonistic and antagonistic networks. (2) A premotor network for directional responses that mediates approach-avoidance output and thereby expresses decision; and (3) Two sets of sensory processing networks, *Incentive* and *Deterrence*, are predicted to integrate afferent sensory inputs and pass them on to the first two networks. Odor learning is presumed to occur in these sensory networks through modulatory feedback (*Reward*) from the activated motor networks. Thus, the network interconnections modify appetitive state, mediate reward, and direct behavioral choice.

*The feeding motor network* itself in *Pleurobranchaea* is a major homeostatic, core processor of foraging decision, manifesting appetitive state in the extent and configuration of its excitation (cf. Figure 2), and thus setting feeding thresholds and assigning stimulus values on the basis of need and incentive. Most recurrent circuit models for categorical choice incorporate leaky integrator modules and recurrent inhibition (cf. Wang, 2008). These qualities are reprised in the dynamic circuitry of the feeding network, its sensory inputs, and its interactions with turning and escape swim networks (Gillette et al., 1982; Jing and Gillette, 1995, 2000, 2003). Its corollary outputs control the approach/avoidance output of the turn network. Sensory inputs, effects of learned odors, and hunger state sum in the excitation state of the feeding network, directly targeting critical identified interneurons, and indirectly excite or inhibit feeding command neurons (Gillette et al., 1982; London and Gillette, 1986; cf. also Gillette, 2008).

*Satiation* (internal state) sums into appetitive state with the effects of sensation and learning. Both satiation and general arousal mechanisms entail serotonin (5-HT), a modulator of the feeding network (Palovcik et al., 1982; Jing and Gillette, 2003). 5-HT from interneurons in the feeding network regulates excitation state and arousal, much like orexin in mammals (Gillette, 2006). 5-HT content in those neurons varies over fourfold with satiation, which is likely reflected in 5-HT output and consequent regulation of feeding network excitability (Hatcher et al., 2008). This may go a long way toward explaining the conservation of donor appetitive state in the isolated CNS.

**Figure 4:**
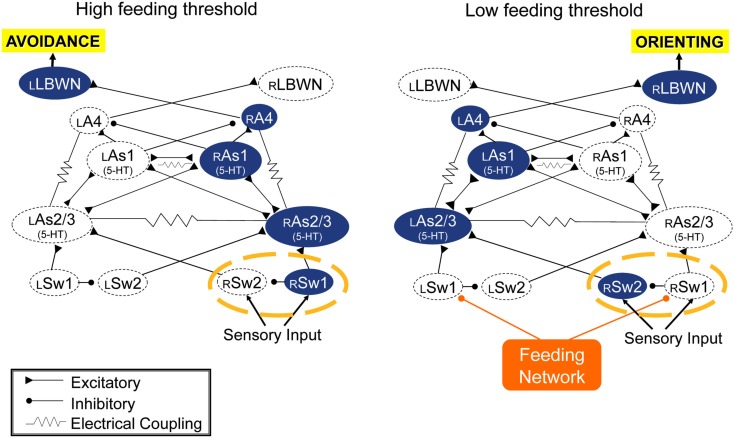
**Simulation suggests that the turn network is default organized for avoidance responses to unilateral sensory inputs (left), and that inputs from the feeding network could reverse responses to orienting (right) via a hypothetical dyad of switch neurons (circled with a broken orange line)**. Double-headed connections indicate reciprocal excitatory connections.

*The turn motor network* computes inputs from the feeding network and sensory inputs from the body, and expresses approach/avoidance decision in the direction and amplitude of its output. It appears by default to be configured for avoidance bhavior to unilateral inputs, but is redirected to orienting by feeding network input. It is expected that sensory inputs determine the computation for turn angle, but orienting turn direction is controlled by the feeding network. When excitation state of the feeding network is low, as in satiated animals, or in absence of appetitive sensory input or when the network is suppressed through learned food avoidance, sensory inputs to the turn network cause avoidance motor output. A simple switch mechanism is shown in Figure 4 that could re-direct sensory input from one side of the turn network to the other, converting avoidance to orienting. This is a hypothesis awaiting test.

*Sensory integration* in prey tracking is to a large extent performed at the animal’s oral (Figure 1), the anterior chemotactile structure where sensory afferents feed into peripheral ganglia (Bicker et al., 1982a,b). Those in turn send integrated information to the CNS in the sensory Large Oral Veil (LOVN) and Tentacle nerves (TN), respectively. Functionally, the oral veil is a composite of mammalian gustatory and olfactory system, with receptors for amino acids (but not sweet or bitter chemicals) to assess nutritive content, and others that must encode odors for associative learning. Turning behavior has been described quantitatively. In prey tracking, *Pleurobranchaea* averages chemotactile stimuli at multiple sites on the oral veil into precise angles of turn, and also uses a simple working memory to optimize the chase (Yafremava et al., 2007). Two relevant findings are: (1) turn direction is affected by appetitive state, so non-hungry animals actively avoid appetitive stimuli; and (2) the angles of avoidance turns induced by noxious stimuli are computed similarly to orienting, only differing in direction. Chemotactile stimuli at the oral veil are encoded for site and amplitude in peripheral ganglia via putative lateral inhibition (Yafremava and Gillette, 2011). The information is transmitted by LOVN and TN to CNS, to be integrated for computing turn angle.

The peripheral ganglia of the oral veil are thought to integrate primary afferent information regarding both stimulus nutritive content and nociception. Specific odor signatures are so far not detected in LOVN and TN activity (unpublished). Thus, it is presently considered that peripheral ganglia may encode the memory of odors for transmission to the CNS in terms of secondary appetence and nociception in the relatively few sensory interneurons (Yafremava and Gillette, 2011) recordable in the nerve responses.

### Network connectivity

**Figure 5:**
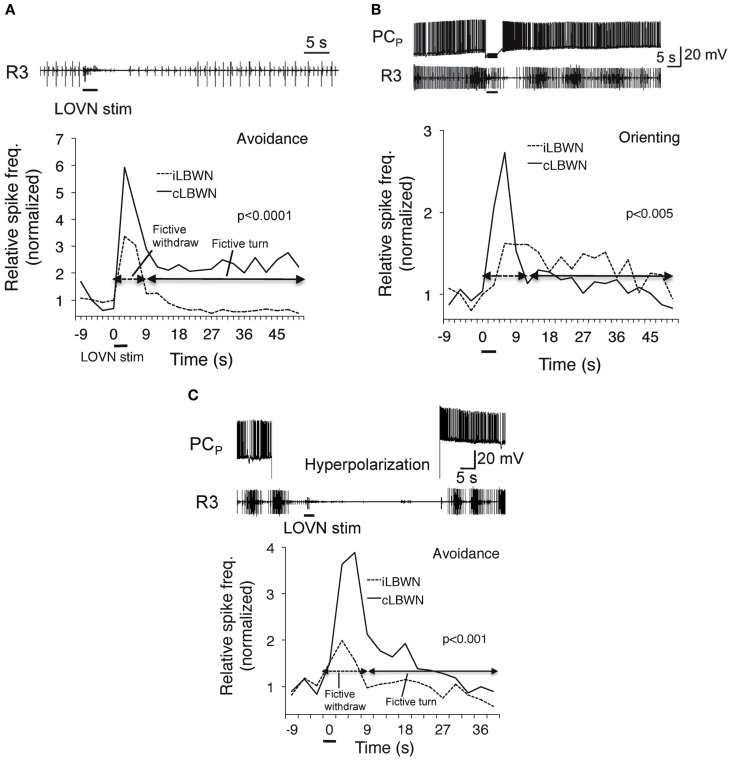
**Dependence of approach/avoidance decision on the excitation state of the feeding motor network**. Fictive avoidance **(A)** was switched to orienting **(B)** following penetration of a feeding command neuron (PCp; Gillette et al., 1982) whose firing induced rhythmic bursting in a feeding nerve (*R*3). Hyperpolarization of PCN **(C)** suppressed fictive feeding and restored avoidance. From Hirayama and Gillette (2012).

Corollary outputs from feeding to turning network reverse the direction of the turn response to unilateral inputs from oral veil through an as yet undetermined switch mechanism (cf. Figure 4). Enhanced activity in the feeding network suppresses the motor output of withdrawal to touch by inhibiting sensory input to withdrawal motor neurons (Kovac and Davis, 1980a). Raising the excitation state of the feeding motor network also suppresses the avoidance turn and promotes the orienting turn (Figure 5). A reciprocal inhibitory pathway from avoidance to feeding, predicted by Kovac and Davis (1980b), has also appeared (unpublished).

It is necessary to postulate existence of reinforcement pathways (*Reward* in Figure 3) to the sensory integrating networks, *Incentive* and *Deterrence*, driven from the goal-directed feeding network and from avoidance to account for odor learning. These would serve to potentiate learning mechanisms for odors and thereby assign them positive or negative values, depending on their association with nutrient reward or punishment in an attack on prey. Perhaps likely mediators are serotonergic neurons, the anterior cerebral cluster (Moroz et al., 1997), embedded in the feeding network and innervating the oral veil, and/or putative dopaminergic neurons histochemically demonstrable in the oral veil (in preparation). Both serotonin and dopamine have functions in molluscan learning (Brembs et al., 2002; Marinesco et al., 2004; Gillette, 2006). Other configurations are possible.

However, the general form of the model is clear. Its advantage is that it may well apply broadly across species. Details of neuropharmacology and internal circuitry of the networks might be expected to vary considerably within the major structure of the model. For instance, mammals, insects, and molluscs differ markedly in roles of dopamine, octopamine, and serotonin in mediating reward (Schwaerzel et al., 2003), but reward mechanisms play similar roles across taxa. The utility of the comparative approach here is not dependent on exact correspondence of neurotransmitter involvement. Similarly, the complicated choices and valuations made by social vertebrates are likely to arise from circuitry concatenated upon the basic neuronal module of cost-benefit decision visible in the simplest model systems.

A preliminary test of the cost-benefit decision model has been done in a computational simulation of foraging and prey choice. The logical relations of the model of Figure 3 are implemented in simple equations representing sensation, appetitive state, orienting and avoidance, and odor learning, and the resulting predator/prey simulation successfully reproduces state- and learning-dependent cost-benefit decisions of the real sea-slug predator. Cyberslug 2.0 is presently available for preview and interactive play at http://www.life.illinois.edu/slugcity/Cyberslug21.html.

### Future directions

What actual benefit is offered by the model of Figure 3 and the resulting simulation? We expect that this type of model forms a basis for a bottom-up approach to cognitive processes higher than *Pleurobranchaea* could ever achieve. The sea-slug is streamlined for simplicity in predation and reproduction, and completely lacking in any of the social graces that normally attend cognitive processes in mammals; its only social behaviors are copulation and (arguably) cannibalism, and its larvae are left to find their own luck with myriad other plankton. Thus, the bare-bones model of the decision process is markedly amenable to *in simulo* experiment with modifications and add-ons that could bring the artificial entities toward social interactions characteristic of functional cognition and consciousness. Some logical add-ons that might be implemented in evolutionarily plausible ways to achieve social characters of higher vertebrates could be territoriality, social hierarchy formation, and altruistic partnering. Here, the building-from-the-bottom-up approach is a potent complement to the top-down, in which the truly intelligent animals are taken apart like one might analyze a complex electronic instrument. Modern computers and communication devices themselves were developed over time from very simple electronic circuits. Indeed, the training of our technicians begins with and builds on those simple circuits. The analogy is obvious for the comparative approach to understanding higher function. We look forward to future efforts in neuroscience and engineering aimed at moving back the borders of this frontier.

Interactions of appetitive state with neural bases of reward, learning, and cognition are also only beginning to be appreciated at the neuronal level (e.g., Tindell et al., 2009) although they have been long regarded as basic to decision. Hebb and Thompson (1954) noted over 60 years ago that “…*cortical or cognitive components in motivation are clearest when we compare the behavior of higher and lower species. Application of a genuine comparative method is essential in the field of motivation as well as of intellectual functions*.” It is timely that the comparative method finds fuller fruition now in parallel studies of self-awareness, cognition, and value assessment in species ranging across nematodes, rotifers, sea-slugs, cephalopods, leeches, arthropods, fish, frogs, birds, rodents, carnivores, and the varied species of primates.

## Conflict of Interest Statement

The authors declare that the research was conducted in the absence of any commercial or financial relationships that could be construed as a potential conflict of interest.

## References

[B1] Bicker G. Davis W. J. Matera E. M. Kovac M. P. Stormo Gipson D. J. (1982a). Chemoreception and mechanoreception in the gastropod mollusc *Pleurobranchaea californica*. I. Extracellular analysis of afferent pathways. J. Comp. Physiol. 149, 221–234 10.1007/BF00619217

[B2] Bicker G. Davis W. J. Matera E. (1982b). Chemoreception and mechanoreception in the gastropod mollusc *Pleurobranchaea californica*. II. Neuroanatomical and intracellular analysis of afferent pathways. J. Comp. Physiol. 149, 235–250 10.1007/BF00619217

[B3] Brembs B. Lorenzetti F. D. Reyes F. D. Baxter D. A. Byrne J. H. (2002). Operant reward learning in Aplysia: neuronal correlates and mechanisms. Science 296, 1706–1709 10.1126/science.1069434 12040200

[B4] Davis W. J. Gillette R. (1978). Neural correlate of behavioral plasticity in command neurons of *Pleurobranchaea*. Science 199, 801–804 10.1126/science.622572 622572

[B5] Davis W. J. Mpitsos G. J. (1971). Behavioral choice and habituation in the marine mollusk *Pleurobranchaea californica*. Z. Vergl. Physiol. 75, 207–232

[B6] Davis W. J. Villet J. Lee D. Rigler M. Gillette R. Prince E. (1980). Selective and differential avoidance learning in the feeding and withdrawal behavior of *Pleurobranchaea californica*. J. Comp. Physiol. 138, 157–165 10.1007/BF00680439

[B7] Gillette R. (2006). Evolution and function in serotonergic systems. Integ. Comp. Biol. 46, 838–846 10.1093/icb/icl024 21672789

[B8] Gillette R. (2008). “Behavioral hierarchies,” in The New Encyclopedia of Neuroscience, ed. Squire L. (Amsterdam: Elsevier).

[B9] Gillette R. Huang R.-C. Hatcher N. Moroz L. L. (2000). Cost-benefit analysis potential in feeding behavior of a predatory snail by integration of hunger, taste, and pain. Proc. Natl. Acad. Sci. U.S.A. 97, 3585–3590 10.1073/pnas.97.7.3585 10737805 PMC16283

[B10] Gillette R. Kovac M. P. Davis W. J. (1982). Control of feeding motor output by paracerebral neurons in the brain of *Pleurobranchaea* *californica*. J. Neurophysiol. 47, 885–908 7086474 10.1152/jn.1982.47.5.885

[B11] Hatcher N. G. Zhang X. Potgieter K. Moroz L. L. Sweedler J. V. Gillette R. (2008). 5-HT and 5-HT-SO_4_, but not 5-HIAA, in single feeding neurons track animal hunger state. J. Neurochem. 104, 1358–1363 10.1111/j.1471-4159.2007.05084.x 18036151 PMC4024471

[B12] Hebb D. O. Thompson W. R. (1954). “The social significance of animal studies,” in Handbook of Social Psychology. ed. Lindzey G. (Cambridge, MA: Addison-Wesley), 532–561

[B13] Hirayama K. Gillette R. (2012). A neuronal network switch for approach/avoidance toggled by appetitive state. Curr. Biol. 22, 118–123 10.1016/j.cub.2011.10.055 22197246 PMC3267890

[B14] Jing J. Gillette R. (1995). Neuronal elements that mediate escape swimming and suppress feeding behavior in the predatory seaslug *Pleurobranchaea*. J. Neurophysiol. 74, 1900–1910 8592183 10.1152/jn.1995.74.5.1900

[B15] Jing J. Gillette R. (2000). Distinct corollary outputs of the escape swim motor network mediate a behavioral switch and excite a distributed serotonergic network in *Pleurobranchaea*. J. Neurophysiol. 83, 1346–1355 10712462 10.1152/jn.2000.83.3.1346

[B16] Jing J. Gillette R. (2003). Directional avoidance turns encoded by single neurons and sustained by multifunctional serotonergic cells. J. Neurosci. 23, 3039–3051 12684491 10.1523/JNEUROSCI.23-07-03039.2003 PMC6742103

[B17] Kovac M. P. Davis W. J. (1980a). Neural mechanism underlying behavioral choice in *Pleurobranchaea*. J. Neurophysiol. 43, 469–487 7381529 10.1152/jn.1980.43.2.469

[B18] Kovac M. P. Davis W. J. (1980b). Reciprocal inhibition between feeding and withdrawal behaviors in *Pleurobranchaea*. J. Comp. Physiol. 139, 77–86 10.1007/BF00666197

[B19] London J. A. Gillette R. (1986). Mechanism for food avoidance learning in the central pattern generator of feeding behavior of *Pleurobranchaea californica*. Proc. Natl. Acad. Sci. U.S.A. 83, 4058–4062 10.1073/pnas.83.11.4058 16593706 PMC323665

[B20] Marinesco S. Kolkman K. E. Carew T. J. (2004). Serotonergic modulation in *Aplysia*. I. Distributed serotonergic network persistently activated by sensitizing stimuli. J. Neurophysiol. 92, 2468–2486 10.1152/jn.00210.2004 15140903

[B21] Moroz L. L. Sudlow L. C. Jing J. Gillette R. (1997). Serotonin-immunoreactivity in peripheral tissues of the opisthobranch molluscs *Pleurobranchaea* *californica* and *Tritonia diomedea*. J. Comp. Neurol. 382, 176–188 10.1002/(SICI)1096-9861(19970602)382:2<176::AID-CNE3>3.0.CO;2-0 9183687

[B22] Mpitsos G. J. Collins S. D. (1978). Learning: rapid aversive conditioning in the gastropod mollusk *Pleurobranchaea*. Science 188, 954–957 1138366 10.1126/science.1138366

[B23] Mpitsos G. J. Cohan C. S. (1986a). Differential Pavlovian conditioning in the mollusc *Pleurobranchaea*. J. Neurobiol. 17, 487–497 10.1002/neu.480170509 3772365

[B24] Mpitsos G. J. Cohan C. S. (1986b). Discriminative behavior and Pavlovian conditioning in the mollusc *Pleurobranchaea*. J. Neurobiol. 17, 469–486 10.1002/neu.480170509 3772364

[B25] Palovcik R. R. Basberg B. A. Ram J. L. (1982). Behavioral state changes induced in *Pleurobranchaea* and *Aplysia* by serotonin. Behav. Neural Biol. 35, 383–394 10.1016/S0163-1047(82)91034-2 7165620

[B26] Schwaerzel M. Monastirioti M. Scholz H. Friggi-Grelin F. Birman S. Heisenberg M. (2003). Dopamine and octopamine differentiate between aversive and appetitive olfactory memories in *Drosophila*. J. Neurosci. 23, 10495–10502 14627633 10.1523/JNEUROSCI.23-33-10495.2003 PMC6740930

[B27] Sherrington C. (1906). The Integrative Action of the Nervous System. New Haven: Yale University Press, 7

[B28] Stuart D. G. (2002). Reflections on spinal reflexes. Adv. Exp. Med. Biol. 508, 249–257 10.1007/978-1-4615-0713-0_30 12171118

[B29] Tindell A. J. Smith K. S. Berridge K. C. Aldridge J. W. (2009). Dynamic computation of incentive salience: “wanting” what was never “liked.” J. Neurosci. 29, 12220–12228 19793980 10.1523/JNEUROSCI.2499-09.2009 PMC2792765

[B30] Wang X.-J. (2008). Decision making in recurrent neuronal circuits. Neuron 60, 213–234 10.1016/j.neuron.2008.09.034 PMC2710297 18957215

[B31] Yafremava L. S. Anthony C. W. Lane L. Campbell J. K. Gillette R. (2007). Orienting and avoidance turning are precisely computed by the predatory sea-slug *Pleurobranchaea californica* McFarland. J. Exp. Biol. 210, 561–569 10.1242/jeb.02697 17267641

[B32] Yafremava L. S. Gillette R. (2011). Putative lateral inhibition in sensory processing for directional turns. J. Neurophysiol. 105, 2885–2890 10.1152/jn.00124.2011 21490281 PMC3118739

